# Screening of co-expressed genes in hypopharyngeal carcinoma with esophageal carcinoma based on RNA sequencing and Clinical Research

**DOI:** 10.1038/s41598-024-64162-w

**Published:** 2024-06-14

**Authors:** Jianing Zhang, Liangyu Zou, Fuxian Tan, Hongmin Wang, Zhenlei Wen, Hongmei Wang, Lianhe Li

**Affiliations:** Department of Otorhinolaryngology Head and Neck Surgery, Central Hospital of Chaoyang, Liaoning, 122000 China

**Keywords:** Bioinformatics analysis, Hypopharyngeal squamous cell carcinoma combined with esophageal squamous cell carcinoma, Nomogram, ROC curve, RNA sequencing, Oncology, Medical research, Diagnostic markers, Head and neck cancer

## Abstract

To explore the hub comorbidity genes and potential pathogenic mechanisms of hypopharyngeal carcinoma with esophageal carcinoma, and evaluate their diagnostic value for hypopharyngeal carcinoma with co-morbid esophageal carcinoma. We performed gene sequencing on tumor tissues from 6 patients with hypopharyngeal squamous cell carcinoma with esophageal squamous cell carcinoma (hereafter referred to as “group A”) and 6 patients with pure hypopharyngeal squamous cell carcinoma (hereafter referred to as “group B”). We analyzed the mechanism of hub genes in the development and progression of hypopharyngeal squamous cell carcinoma with esophageal squamous cell carcinoma through bioinformatics, and constructed an ROC curve and Nomogram prediction model to analyze the value of hub genes in clinical diagnosis and treatment. 44,876 genes were sequenced in 6 patients with group A and 6 patients with group B. Among them, 76 genes showed significant statistical differences between the group A and the group B.47 genes were expressed lower in the group A than in the group B, and 29 genes were expressed higher. The top five hub genes were GABRG2, CACNA1A, CNTNAP2, NOS1, and SCN4B. GABRG2, CNTNAP2, and SCN4B in the hub genes have high diagnostic value in determining whether hypopharyngeal carcinoma patients have combined esophageal carcinoma (AUC: 0.944, 0.944, 0.972). These genes could possibly be used as potential molecular markers for assessing the risk of co-morbidity of hypopharyngeal carcinoma combined with esophageal carcinoma.

## Introduction

The HPSCC (hypopharyngeal squamous cell carcinoma) is one of the head and neck tumors with high malignancy. Its incidence rate is about 3–5%^1^ in head and neck malignancies. Research shows that the prognosis of hypopharyngeal carcinoma is poor, with a recurrence median survival period of 6–7 months and a 5-year recurrence-free survival rate of 30–40%^2^. The Second Primary Malignancy (SPM) is an independent prognostic factor affecting the survival rate of hypopharyngeal carcinoma, significantly increasing the risk of death in patients with nasopharyngeal carcinoma^3^. The esophagus is the most common site of SPMs in patients with head and neck squamous cell carcinoma, with an incidence rate of up to 30% and a risk of 28.6 times higher than that of the normal population^4^.

Hypopharyngeal-esophageal carcinomas have no obvious symptoms at the early stage of the disease, because of the special anatomical position of the region, electronic gastroscopy often passes through this region quickly to reduce the pain of the patients, and laryngoscopy is often not able to peep into the esophagus, so it is difficult to diagnose in the early stage, and the diagnosis is often progressed to the middle and late stages when confirmed. According to the recommendations of the guidelines for the diagnosis and treatment of multiple primary cancers of the hypopharynx-esophagus^4^, for patients with synchronous hypopharyngeal carcinoma combined with esophageal carcinoma, the comprehensive treatment model of surgical treatment followed by postoperative chemoradiotherapy is an ideal treatment method. However, the complications of surgical treatment, such as gastric emptying dysfunction, gastroesophageal anastomotic leakage, anastomotic stenosis, or pleural effusion, seriously affect the quality of life of patients^5^. For patients with metachronous hypopharyngeal carcinoma combined with esophageal carcinoma, the treatment options for secondary carcinoma are always limited by thetreatment of the first primary carcinoma, resulting in long treatment cycles andpoor efficacy for patients. Currently, there are no clear reports on the positive efficacy of targeted therapy and immunotherapy for multiple primary carcinomain combination. Gene sequencing is a method for analyzing genome sequences, enabling genetic evolution analysis and prediction of important trait candidate genes, as well as analyzing the base sequence of specific DNA fragments. Identifying hub co-disease genes and their possible regulatory molecular mechanisms in hypopharyngeal carcinoma, and esophageal carcinoma, patients at the molecular level through gene sequencing and bioinformatics analysis is of great significance for early diagnosis, targeted drug development, and individualized treatment of patients with hypopharyngeal carcinoma combined with esophageal carcinoma.

## Methods

1. Experimental materials

This study included six cases of hypopharyngeal carcinoma combined with esophageal carcinoma in patients and six patients with hypopharyngeal carcinoma. Postoperative pathology confirmed squamous cell carcinoma of the hypopharynx and esophagus. The age range of all patients was 18–80 years old, and their clinical data were complete. All patients had signed relevant informed consent before surgery. Patients with previous primary tumor history other than hypopharyngeal and esophageal carcinoma, or previousesophageal or hypopharyngeal surgery, radiotherapy, or chemotherapy were excluded. Before taking specimens, the surgeon and pathologist communicated with each other about the sampling site and size of the specimen. Pathological specimens were taken from the hypopharyngeal cancer section. The central part of the tumor without necrosis and infection was selected for tissue specimen collection, and it was ensured that the tumor after being taken would not affect the patient's pathological diagnosis. During the operation, after the specimen was removed from the body, it was rinsed twice with saline. Immediately cut two pieces of non-necrotic tumor tissue with a size of 0.5 cm^3^, and placed them in EP tubes. Within 30 min, transfer them to the corresponding position of the – 80 ℃ refrigerator for preservation. The pathological diagnosis of the tissue was made by two experienced chief pathologists from the Department of Pathology of Chaoyang Central Hospital by the WHO diagnostic criteria, and it was ensured that the tumor cell ratio of the tumor samples in this study was greater than 80%. The patient’s TNM staging and clinical staging standards were based on the 8th edition of the American Joint Committee on Carcinoma (AJCC) TNM Staging Manual in 2017. This study was approved by the Ethics Committee of Chaoyang Hospital with ethical approval number [2022] 30, and all patients provided informed written consent. Patients clinical information sheet in Table 1.

**Table 1:** Patient clinical information sheet.

Patient ID	Age (years)	Sex	Cigarettes per day	Years of smoking	Alcohol consumption each day (g)	Years of alcohol consumption (year)	TNM stage
A1	57	Male	15–20	70Y	500	20Y	T4N1M0
A2	59	Male	20–30	40Y	100	10Y	T4N0M0
A3	79	Male	5–20	60Y	350	40Y	T2N0M0
A4	54	Male	10–20	30Y	-	30Y	T4N1M0
A5	64	Male	20–25	40Y	70	20Y	T4N1M0
A6	51	Male	20–25	30Y	150	30Y	T4N2M0
B1	69	Male	20–22	40Y	500	40Y	T3N1M0
B2	71	Male	18–20	40Y	200	40Y	T3N1M0
B3	50	Male	20–30	40Y	500	40Y	T4N2M0
B4	67	Male	17–20	50Y	5	40Y	T3N0M0
B5	55	Male	10–20	30Y	10	30Y	T3N2M0
B6	65	Male	20–40	10Y	100	20Y	T3N2M0

2. RNA sequencing

Detect the purity of the sample and the concentration and integrity of the RNA sample. Take 1–3 μg total RNA from each sample as the starting material to construct a transcriptome sequencing library. According to the instructions of the library preparation kit, select different index tags to construct the library and use Qubit 3.0 for preliminary quantification. Dilute the library to 1 ng/ul, detect the insert size of the library, and use the Illumina platform for sequencing. Run the PE150 sequencing strategy to obtain 150 bp paired-end sequencing reads. The Raw Data is stored in FASTQ file format, removing reads with adapters, uncertain base information, and low-quality reads (Qphred <= 20 base number accounting for more than 50% of the length of the entire read).

3. Bioinformatics analysis of differential genes

The GO (Gene Ontology) database is an ontology widely used in the field of bioinformatics. It covers three aspects of biology: cellular components, molecular function, and biological processes. The functional enrichment analysis method annotates the selected genes based on the GO database to obtain all the functions involved in the genes and then uses Fisher’s exact test and multiple comparison tests to calculate the significance level and false discovery rate (FDR) for each function. This allows the screening of significant functions embodied by genes, with the criterion of significance screening being *p* < 0.05.

KEGG (Kyoto Encyclopedia of Genes and Genomes) is a database that integrates genomic, chemical, and system function information. It is a database for systematically analyzing the relationships between genes (and their encoded products), gene functions, and genomic information. It helps researchers study genes and expression information as a whole network. Significance screening criteria: *p* < 0.05.

Map the differentially expressed gene set to the STRING database (STRING: functional protein association networks, http://cn.string-db.org) to construct a protein interaction network of differentially expressed genes. Import the differentially expressed genes into the STRING database for analysis, and import the output file into Cytoscape for visualization. Using the cytoHubba plugin in Cytoscape, calculate the hub genes based on degree.

4. Use R software packages such as ggplot2, Survival, timeROC, limma, etc. for analysis. Import the hub genes into relevant files and use the aforementioned packages to calculate the ROC curve and nomogram of the hub genes.
5. PCR Verification of gene selection

The six differentially expressed genes with significant differences and relatively high expression levels in the 12 groups of samples were selected for PCR detection: LINC00470, CYP2B7P, NKX2-8, CADM3, MIPOL1 and ZFP82. Gene sequencing and PCR-related reagents are shown in Table 2. Gene and primer information of PCR in Tables 3 and 4. Approximately 100 mg of tumor tissue from the cryopreservation tube was cut with sterile scissors and placed in an RNase-free centrifuge tube. Two steel balls were added, followed by 1000 μL of tissue lysate (Trizol). The tube was centrifuged in a high-channel tissue homogenizer, and the total RNA was extracted using the centrifugation solution and reverse transcribed. After diluting the reverse transcribed cDNA, real-time quantitative PCR was performed using SYBR Green I for gene expression analysis. The relative expression was calculated using the 2^−△△Ct^ method. GraphPad Prism 9.0 statistical software was used to process the data for graphing, and the independent samples *t*-test was used for measures that conformed to normal distribution, and the nonparametric test was used for measures that were not normally distributed, with *p* < 0.05 being statistically significant.

**Table 2:** Gene sequencing and PCR-related reagents.

Gene sequencing and PCR-related reagents	
RNA extraction reagent	brand Invitrogen TRIZOL Reagent, item number Cat No.15596–018
Library Preparation Kit	VAHTS Universal V6 RNA seq Library Prep Kit for Illumina^®^ (NR604-01/02)
Sequencing reagent	NovaSeq ™ X 10B RGB CART 300 CYLE; NovaSeq 6000 S4 Flow Cell
	DNBSEQ-T7RS High throughput Sequencing Kit (FCL PE150) V2.0
Test sample purity	NanoPhotometer ® Spectrophotometer (IMPLEN, CA, USA)
Testing the concentration and integrity of RNA samples	Agilent Technologies, CA, USA, Agilent 2100 RNA Nano 6000 Assay Kit
Reverse Transcription Kit	Transgen Reverse Transcription Kit,AE-311 EasyScript One Step gDNA Removal and cDNA Synthesis SuperMix
Centrifuge solution	Centrifuge solution: Kangwei Century Ultra RNA Kit (DNase I), CW0597S
PCR reagent kit	TransGen Green qPCR SuperMix (product number: AQ131-01), all gold,The detection machine is LightCycler96 (Roche, Swiss)

**Table 3:** Gene information of PCR.

Gene	Log2FoldChange	padj	Up/down	Max FPKM
LINC00470	4.631422741	0.000563954	Up	1.055446426
CYP2B7P	5.85196451	0.005584158	Up	11.21620163
NKX2-8	4.599889396	0.008341835	Up	45.74681219
CADM3	− 3.794842159	0.023661956	Down	7.10985851
MIPOL1	3.354907238	0.024823833	Up	1.684702513
ZFP82	− 2.653958335	0.040049932	Down	0.656817591

**Table 4:** Gene primer information of PCR.

Primer	Primer sequence: 5′–3′	Primer length	Product length
GAPDH-F	GAAGGTGAAGGTCGGAGTCAA	21	229
GAPDH-R	CTGGAAGATGGTGATGGGATTT	22	
LINC00470-F2	GCCAGATGCTTGCTTTTACAG	21	95
LINC00470-R2	CTCTCCCAGACACCCATGTT	20	
CYP2B7P-F2	CCTTAGGGAAGCGGATTTGT	20	118
CYP2B7P-R2	TCGATGTCTTCAGGAGCCAC	20	
NKX2-8-F2	GCCAGCCCCAGAGTTAGATT	20	141
NKX2-8-R2	GGACCCAACCCCACACATTT	20	
CADM3-F2	GGTGCTCAAGTGCCAAGTGA	20	138
CADM3-R2	TCGTGGGGCGTAGAGGTAAC	20	
MIPOL1-F2	GTTGGAAAGGCTGGTGGATGT	21	100
MIPOL1-R2	TGATCGCTTCCCCAGACTGT	20	
ZFP82-F1	CCTCTGTGGACTCGGTGCTT	20	85
ZFP82-R1	TCGAAGGGCCATGGTATAGA	20	

### Ethical approval

The study was approved by the Ethics Committee of Chaoyang Downtown Hospital and informed written consent was obtained from all patients. Primary tumors and adjacent normal tissues of HPSCC patients were obtained according to the Declaration of Helsinki.

## Results

**Figure 1:**
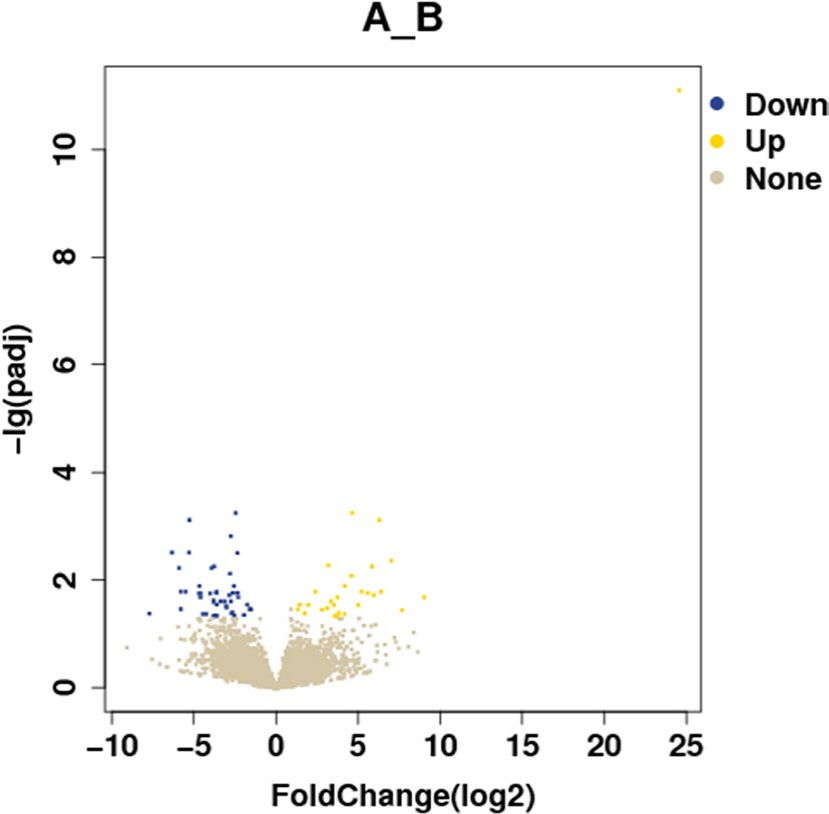
Differential expression genes volcano diagram.

1. Results of differentially expressed genes: A total of 44,876 genes were screened and 76 genes showed significant statistical differences between the hypopharyngeal carcinoma with esophageal carcinomagroup, and the hypopharyngeal carcinoma group (Screening Principle|log2(FoldChange)|> 1 *p* < 0.05). Among them, 47 genes were expressed lower in the hypopharyngeal carcinoma with the esophageal carcinoma group than in the hypopharyngeal carcinoma group, and 29 genes were expressed higher (Table 5). The sequencing results are shown in Table 6. The results of the differential gene visualization volcano map and heatmap are shown in Figs. 1 and 2. Group A is hypopharyngeal cancer combined with esophageal cancer, Group B is hypopharyngeal cancer.

**Table 5:** Results for 76 DEGs.

GeneName	Up/Down	pval	FDR	Log2FoldChange
ENSG00000236531	Up	2.98E-16	7.96E-12	24.55133199
ENSG00000251023	Up	2.92E-05	0.020912533	9.035919555
MUC13	Up	8.47E-05	0.036441439	7.696235091
LINC00648	Up	1.64E-06	0.004363954	7.032933442
ALOX15P2	Up	1.59E-05	0.01645556	6.406212449
GABRG2	Up	1.39E-07	0.000761251	6.296712481
ENSG00000279281	Up	2.52E-05	0.019062849	5.951622839
CYP2B7P	Up	2.59E-06	0.005584158	5.85196451
ENSG00000265417	Up	1.95E-05	0.017257452	5.578505146
PRR9	Up	1.30E-05	0.01645556	5.220053628
ENSG00000280424	Up	5.63E-05	0.029064299	5.010304659
LINC00470	Up	6.34E-08	0.000563954	4.631422741
NKX2-8	Up	5.32E-06	0.008341835	4.599889396
FEZF1-AS1	Up	9.34E-06	0.012981409	4.181809779
FEZF1	Up	0.00011207	0.042708337	4.158953538
CACNA1A	Up	9.66E-05	0.040270173	3.821843586
PSG5	Up	2.99E-05	0.020912533	3.753728256
ENSG00000253851	Up	0.000135302	0.046874378	3.750280491
HTR2C	Up	0.000130645	0.046467743	3.59507786
ENSG00000285571	Up	5.65E-05	0.029064299	3.524236953
MIPOL1	Up	4.19E-05	0.024823833	3.354907238
PCDHB8	Up	2.20E-06	0.005345804	3.202296051
ENSG00000189229	Up	6.86E-05	0.033294575	3.119574683
NECTIN3	Up	8.15E-05	0.035655971	2.790739071
FMO4	Up	1.36E-05	0.01645556	2.403152832
ENSG00000249626	Up	5.67E-05	0.029064299	1.97325239
ENSG00000259001	Up	0.000101574	0.041685871	1.75534513
NUSAP1	Up	5.27E-05	0.028670667	1.445466883
ENSG00000285920	Up	7.72E-05	0.034834716	1.319917483
CACNG5	Down	0.000105822	0.042132813	− 7.722998244
PLA2G2A	Down	8.75E-07	0.003062798	− 6.332922068
ENSG00000287575	Down	3.37E-06	0.005988015	− 5.901729557
PLD5	Down	7.40E-05	0.034665201	− 5.801617394
LINC02843	Down	1.60E-05	0.01645556	− 5.789406515
ENSG00000279965	Down	1.55E-05	0.01645556	− 5.503187187
NOS1	Down	9.19E-07	0.003062798	− 5.303684379
PKD1L2	Down	1.43E-07	0.000761251	− 5.263329873
SERPINA11	Down	2.13E-05	0.017673768	− 4.67768115
ZBTB16	Down	9.57E-06	0.012981409	− 4.675799926
LINC00392	Down	3.06E-05	0.020912533	− 4.602911762
CPA5	Down	2.23E-05	0.017673768	− 4.589315155
C1orf68	Down	0.000111506	0.042708337	− 4.458829326
KPRP	Down	0.000111966	0.042708337	− 4.243362696
PNMA8A	Down	2.25E-05	0.017673768	− 4.014166687
ENSG00000229588	Down	3.27E-06	0.005988015	− 3.923284642
ESPNL	Down	0.000132911	0.046651693	− 3.81388858
IGSF9B	Down	4.39E-05	0.025443124	− 3.803978863
CADM3	Down	3.64E-05	0.023661956	− 3.794842159
PLA2G3	Down	2.72E-06	0.005584158	− 3.765462961
LMAN1L	Down	0.000123417	0.045099438	− 3.707735018
LINC00551	Down	1.91E-05	0.017257452	-3.628858571
ADGRD1	Down	1.56E-05	0.01645556	− 3.618057724
CHIT1	Down	5.21E-05	0.028670667	− 3.611374739
TSKS	Down	0.000127662	0.046020479	− 3.596630021
ENSG00000236886	Down	4.01E-05	0.024823833	− 3.362557466
CNTNAP2	Down	4.18E-05	0.024823833	− 3.113909415
CLDN5	Down	6.02E-05	0.030275801	− 3.043814159
TREML1	Down	6.59E-05	0.03257402	− 3.018964258
ADAMTSL1	Down	2.57E-05	0.019062849	− 2.839166762
PAPPA	Down	4.54E-06	0.007561882	− 2.800354625
SCN4B	Down	3.44E-07	0.001531054	− 2.755681424
PHF24	Down	3.96E-05	0.024823833	− 2.737859044
MYO3B	Down	0.000103273	0.041741135	− 2.682240432
ZFP82	Down	9.46E-05	0.040049932	− 2.653958335
ENSG00000225216	Down	2.01E-05	0.017257452	− 2.64235609
NRP2	Down	9.73E-06	0.012981409	− 2.571128417
KCNN1	Down	0.000123226	0.045099438	− 2.542804063
LINC01127	Down	4.57E-08	0.000563954	− 2.465281281
FAM78B	Down	1.94E-05	0.017257452	− 2.383252551
TNNT2	Down	1.05E-06	0.003126716	− 2.351433052
GCNT4	Down	1.76E-05	0.017257452	− 2.341256979
SLC16A6	Down	3.15E-05	0.020989758	− 2.30665346
ENSG00000241352	Down	0.000117797	0.044258525	− 1.943347424
ZNF623	Down	5.13E-05	0.028670667	− 1.753708108
RASSF1-AS1	Down	7.41E-05	0.034665201	− 1.623472417
ADCY7	Down	7.84E-05	0.034834716	− 1.513106508

**Table 6:** Sequencing results.

Sample	A1	A2	A3	A4	A5	A6	B1	B2	B3	B4	B5	B6
Raw Reads Number	4.89E + 07	4.65E + 07	3.90E + 07	4.89E + 07	4.94E + 07	4.76E + 07	4.79E + 07	4.83E + 07	4.86E + 07	4.90E + 07	4.93E + 07	4.97E + 07
Raw Bases Number	7.33E + 09	6.97E + 09	5.84E + 09	7.34E + 09	7.41E + 09	7.13E + 09	7.19E + 09	7.24E + 09	7.29E + 09	7.34E + 09	7.40E + 09	7.45E + 09
Clean Reads Number	4.71E + 07	4.39E + 07	3.73E + 07	4.75E + 07	4.70E + 07	4.56E + 07	4.60E + 07	4.64E + 07	4.67E + 07	4.71E + 07	4.74E + 07	4.78E + 07
Clean Reads Rate (%)	96.31	94.48	95.72	97.18	95.26	96.42	92.47	93.2	89.69	96.68	91.79	85.73
Clean Bases Number	7.06E + 09	6.59E + 09	5.59E + 09	7.13E + 09	7.06E + 09	7.17E + 09	6.70E + 09	6.75E + 09	6.97E + 09	6.79E + 09	6.65E + 09	6.51E + 09
Adapter Polluted Reads Rate (%)	3.69	5.46	4.2	2.81	4.74	3.58	7.53	6.8	10.31	3.31	8.12	14.27
Raw Q30 Bases Rate (%)	94.13	92.88	92.38	95.2	94.09	93.46	92.14	94.17	94.9	94.84	93.6	95.72
Clean Q30 Bases Rate (%)	94.02	92.72	92.19	95.14	93.94	93.34	91.79	93.94	94.64	94.76	93.36	95.39

Raw Reads Number: The number of Reads that were originally downloaded.

Raw Bases Number: The number of bases in the original down-loading sequence.

Clean Reads Number: The number of high-quality Reads obtained after filtering.

Clean Reads Rate (%): The proportion of high-quality sequences obtained after filtering out low-quality reads. The higher this value, the better the sequencing quality or library quality.

Clean Bases Number: The number of bases in the filtered high-quality sequence.

Adapter Polluted Reads Rate (%): the proportion of removed reads contaminated by adapters to the total raw reads.

Raw Q30 Bases Rate (%): The proportion of bases with a sequencing quality value greater than 30 (with an error rate of less than 0.1%) in Raw Reads, as a percentage of the total bases (Raw Reads).

Clean Q30 Bases Rate (%): The proportion of bases with a sequencing quality value greater than 30 (error rate less than 0.1%) in Clean Reads, out of the total bases (Clean Reads).

A1–A6 hypopharyngeal carcinoma with esophageal carcinoma.

B1–B6 hypopharyngeal carcinoma.

**Figure 2:**
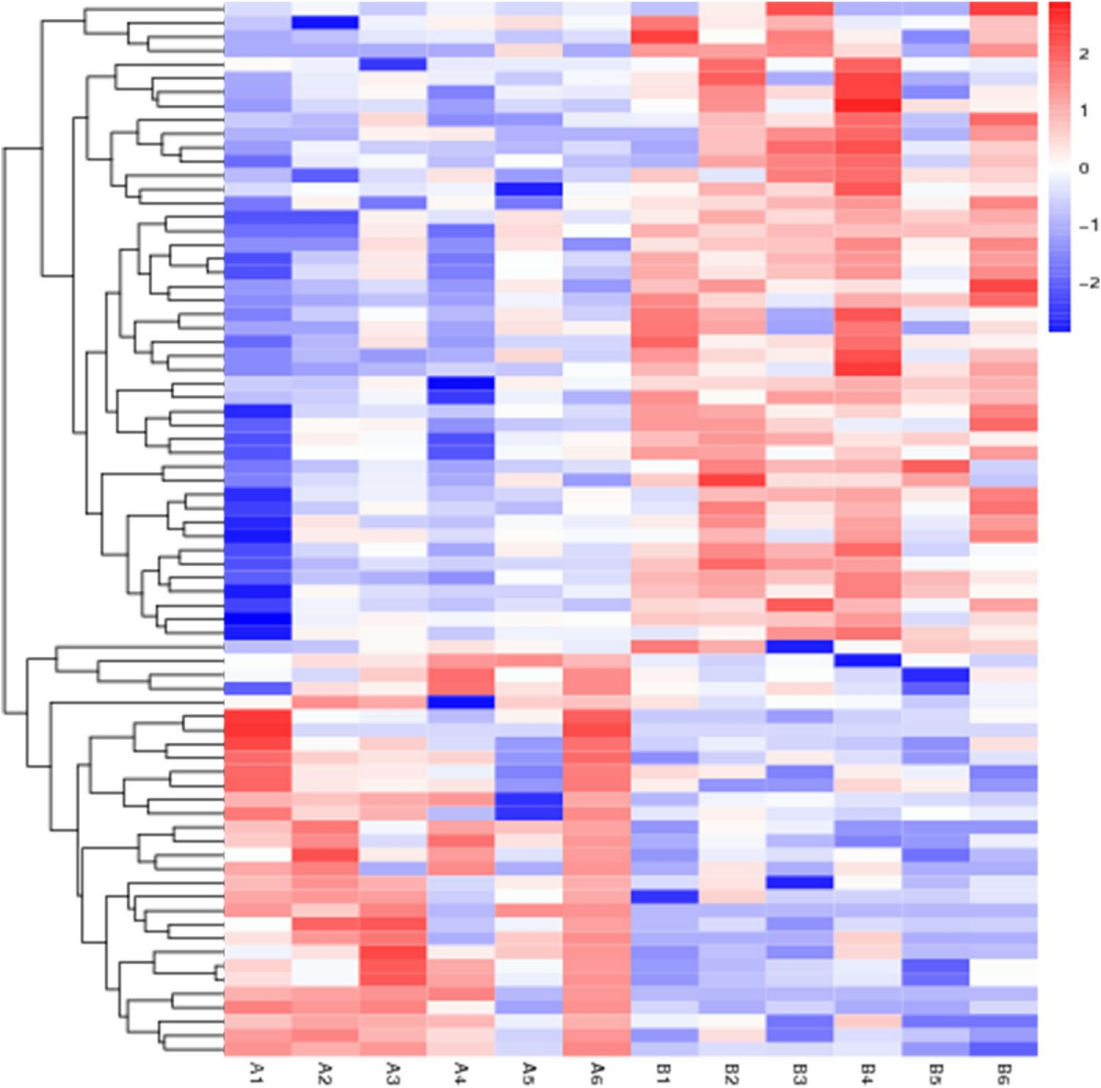
Differential expression genes heatmap.
2. GO analysis results: In GO data analysis, we found that 76 differentially expressed proteins were significantly enriched in 275 GO terms (*p* < 0.05), including 45 terms related to cellular components (GO-CC). Differentially expressed proteins mainly participate in the formation of cell components such as the transporter complex transmembrane transporter complex, cation channel complex, ion channel complex etal. There are 190 items related to biological processes (GO-BP), such as calcium channel activity, cation channel activity, voltage − gated channel activity, transporter activity et al. And 40 related to molecular function (GO-MF), include gamma − aminobutyric acid signaling pathway, calcium ion transmembrane transport, cell adhesion molecules et al. The GO results are shown in Table 7 and Fig. 3.

**Table 7:** GO results.

Ontology	ID	Description	GeneRatio	BgRatio	pvalue	geneID	Count
BP	GO:0,007,157	Heterophilic cell–cell adhesion via plasma membrane cell adhesion molecules	3/43	51/18,903	0.000211591	CADM3/NECTIN3/PSG5	3
BP	GO:0,098,742	Cell–cell adhesion via plasma-membrane adhesion molecules	5/43	282/18,903	0.000431686	PCDHB8/CADM3/NECTIN3/CLDN5/PSG5	5
BP	GO:0,034,374	Low-density lipoprotein particle remodeling	2/43	16/18,903	0.000594387	PLA2G3/PLA2G2A	2
BP	GO:0,010,744	Positive regulation of macrophage derived foam cell differentiation	2/43	19/18,903	0.000843341	PLA2G3/PLA2G2A	2
BP	GO:0,070,588	Calcium ion transmembrane transport	5/43	345/18,903	0.001071674	CACNG5/NOS1/CACNA1A/HTR2C/PKD1L2	5
BP	GO:0,042,178	Xenobiotic catabolic process	2/43	26/18,903	0.001586731	FMO4/NOS1	2
BP	GO:0,007,214	Gamma-aminobutyric acid signaling pathway	2/43	28/18,903	0.001840175	GABRG2/PHF24	2
BP	GO:0,046,337	Phosphatidylethanolamine metabolic process	2/43	28/18,903	0.001840175	PLA2G3/PLA2G2A	2
BP	GO:0,034,368	Protein-lipid complex remodeling	2/43	30/18,903	0.002111564	PLA2G3/PLA2G2A	2
BP	GO:0,034,369	Plasma lipoprotein particle remodeling	2/43	30/18,903	0.002111564	PLA2G3/PLA2G2A	2
CC	GO:0,099,699	Integral component of synaptic membrane	5/45	155/19,869	2.57E-05	CACNG5/GABRG2/NRP2/CADM3/NECTIN3	5
CC	GO:0,099,240	Intrinsic component of synaptic membrane	5/45	166/19,869	3.57E-05	CACNG5/GABRG2/NRP2/CADM3/NECTIN3	5
CC	GO:0,034,702	Ion channel complex	6/45	296/19,869	5.19E-05	CACNG5/KCNN1/GABRG2/CACNA1A/CNTNAP2/SCN4B	6
CC	GO:0,034,703	Cation channel complex	5/45	222/19,869	0.000141328	CACNG5/KCNN1/CACNA1A/CNTNAP2/SCN4B	5
CC	GO:0,099,055	Integral component of postsynaptic membrane	4/45	118/19,869	0.000145928	CACNG5/GABRG2/NRP2/NECTIN3	4
CC	GO:0,098,936	Intrinsic component of postsynaptic membrane	4/45	123/19,869	0.000171226	CACNG5/GABRG2/NRP2/NECTIN3	4
CC	GO:0,097,060	Synaptic membrane	6/45	378/19,869	0.000198301	CACNG5/IGSF9B/GABRG2/NRP2/CADM3/NECTIN3	6
CC	GO:1,902,495	Transmembrane transporter complex	6/45	379/19,869	0.000201151	CACNG5/KCNN1/GABRG2/CACNA1A/CNTNAP2/SCN4B	6
CC	GO:1,990,351	Transporter complex	6/45	405/19,869	0.000287389	CACNG5/KCNN1/GABRG2/CACNA1A/CNTNAP2/SCN4B	6
CC	GO:0,045,211	Postsynaptic membrane	5/45	269/19,869	0.000343928	CACNG5/IGSF9B/GABRG2/NRP2/NECTIN3	5
MF	GO:0,047,498	Calcium-dependent phospholipase A2 activity	2/45	16/18,432	0.000684337	PLA2G3/PLA2G2A	2
MF	GO:0,005,216	Ion channel activity	6/45	446/18,432	0.000710631	CACNG5/KCNN1/GABRG2/CACNA1A/PKD1L2/SCN4B	6
MF	GO:0,015,267	Channel activity	6/45	494/18,432	0.001206382	CACNG5/KCNN1/GABRG2/CACNA1A/PKD1L2/SCN4B	6
MF	GO:0,022,803	Passive transmembrane transporter activity	6/45	495/18,432	0.001218964	CACNG5/KCNN1/GABRG2/CACNA1A/PKD1L2/SCN4B	6
MF	GO:0,022,836	Gated channel activity	5/45	341/18,432	0.001401223	CACNG5/KCNN1/GABRG2/CACNA1A/SCN4B	5
MF	GO:0,005,244	Voltage-gated ion channel activity	4/45	201/18,432	0.001441541	CACNG5/KCNN1/CACNA1A/SCN4B	4
MF	GO:0,022,832	Voltage-gated channel activity	4/45	201/18,432	0.001441541	CACNG5/KCNN1/CACNA1A/SCN4B	4
MF	GO:0,005,261	Cation channel activity	5/45	346/18,432	0.001494113	CACNG5/KCNN1/CACNA1A/PKD1L2/SCN4B	5
MF	GO:0,005,262	Calcium channel activity	3/45	119/18,432	0.003055839	CACNG5/CACNA1A/PKD1L2	3
MF	GO:0,004,623	Phospholipase A2 activity	2/45	36/18,432	0.003483035	PLA2G3/PLA2G2A	2

*BP* biological processes, *CC* cellular components, *MF* molecular functions.

**Figure 3:**
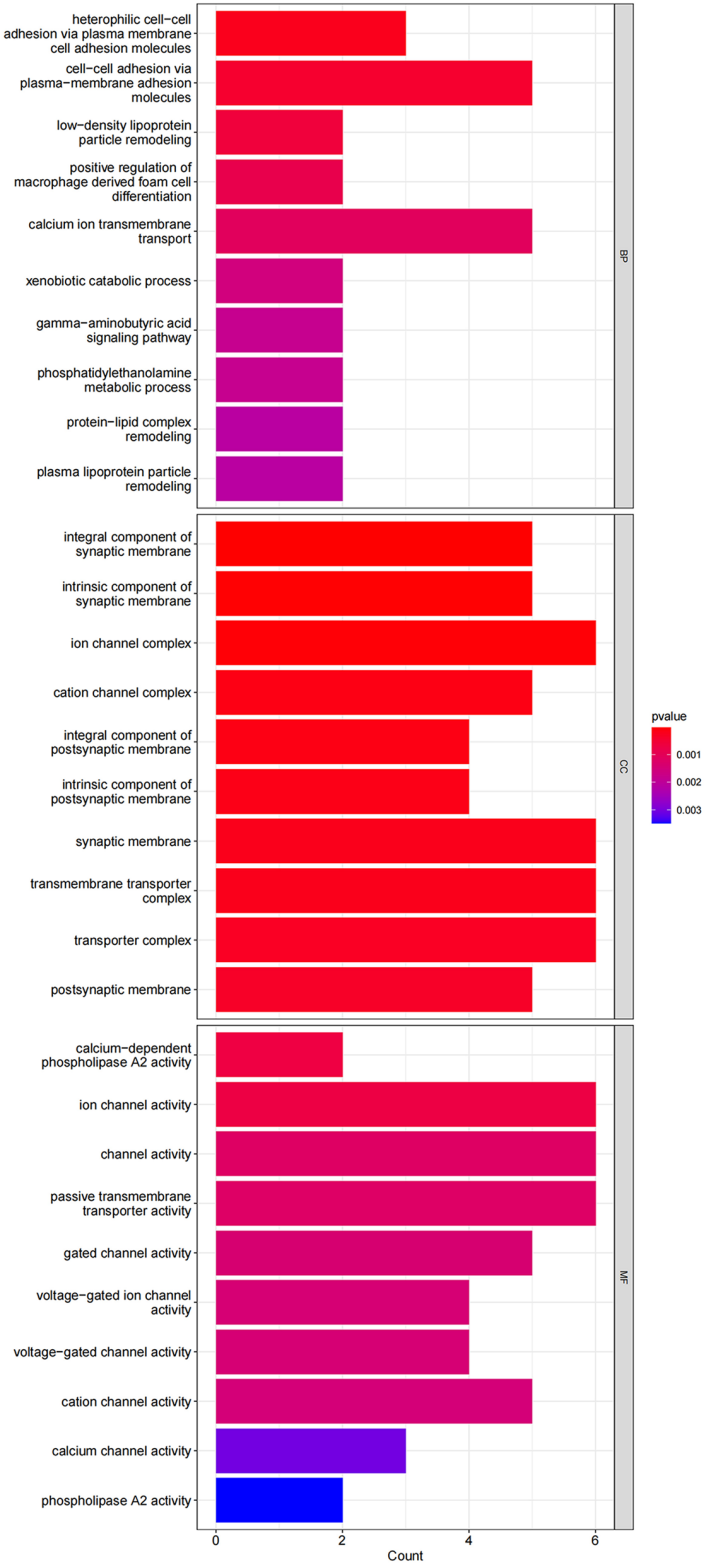
GO result bar chart, showing the top 30 most significant GO items for BP, CC, and MF.
3. KEGG analysis results: KEGG analysis was performed on 76 differentially expressed genes, and KEGG enrichment results were screened using a *p* < 0.05. A total of 29 signaling pathways were identified. The results showed that the differentially expressed genes were mainly involved in carcinoma-related pathways, including cell adhesion molecules, GABAergic synapses, calcium signaling pathways, α-linolenic acid metabolism, and linoleic acid metabolism. The KEGG results are shown in Table 8 and Fig. 4.

**Table 8:** KEGG results.

Description	BgRatio	pvalue	geneID	Count
Adrenergic signaling in cardiomyocytes	154/8579	0.000677676	CACNG5/TNNT2/ADCY7/SCN4B	4
Cell adhesion molecules	158/8579	0.000746296	CADM3/CNTNAP2/NECTIN3/CLDN5	4
GABAergic synapse	89/8579	0.001644905	GABRG2/ADCY7/CACNA1A	3
Morphine addiction	91/8579	0.0017535	GABRG2/ADCY7/CACNA1A	3
Dilated cardiomyopathy	96/8579	0.002044384	CACNG5/TNNT2/ADCY7	3
alpha-Linolenic acid metabolism	26/8579	0.002148855	PLA2G3/PLA2G2A	2
Pancreatic secretion	102/8579	0.002431149	PLA2G3/ADCY7/PLA2G2A	3
Linoleic acid metabolism	30/8579	0.002857483	PLA2G3/PLA2G2A	2
Calcium signaling pathway	253/8579	0.004203071	NOS1/ADCY7/CACNA1A/HTR2C	4
Nicotine addiction	40/8579	0.005041061	GABRG2/CACNA1A	2
Vascular smooth muscle contraction	134/8579	0.005250139	PLA2G3/ADCY7/PLA2G2A	3
Fat digestion and absorption	43/8579	0.005807599	PLA2G3/PLA2G2A	2
Retrograde endocannabinoid signaling	148/8579	0.006917854	GABRG2/ADCY7/CACNA1A	3
Ether lipid metabolism	50/8579	0.007789416	PLA2G3/PLA2G2A	2
Long-term depression	60/8579	0.011073689	NOS1/CACNA1A	2
Arachidonic acid metabolism	61/8579	0.011430512	PLA2G3/PLA2G2A	2
Insulin secretion	86/8579	0.021923953	KCNN1/ADCY7	2
Cardiac muscle contraction	87/8579	0.02240356	CACNG5/TNNT2	2
Gap junction	88/8579	0.022887544	ADCY7/HTR2C	2
Hypertrophic cardiomyopathy	90/8579	0.023868545	CACNG5/TNNT2	2
Salivary secretion	93/8579	0.025372259	NOS1/ADCY7	2
Circadian entrainment	97/8579	0.027436318	NOS1/ADCY7	2
Inflammatory mediator regulation of TRP channels	98/8579	0.027962728	ADCY7/HTR2C	2
Glycerophospholipid metabolism	99/8579	0.028493247	PLA2G3/PLA2G2A	2
Cholinergic synapse	113/8579	0.036338937	ADCY7/CACNA1A	2
Glutamatergic synapse	115/8579	0.037521417	ADCY7/CACNA1A	2
Serotonergic synapse	115/8579	0.037521417	CACNA1A/HTR2C	2
Taurine and hypotaurine metabolism	16/8579	0.042079694	FMO4	1
Relaxin signaling pathway	129/8579	0.046205357	NOS1/ADCY7	2

**Figure 4:**
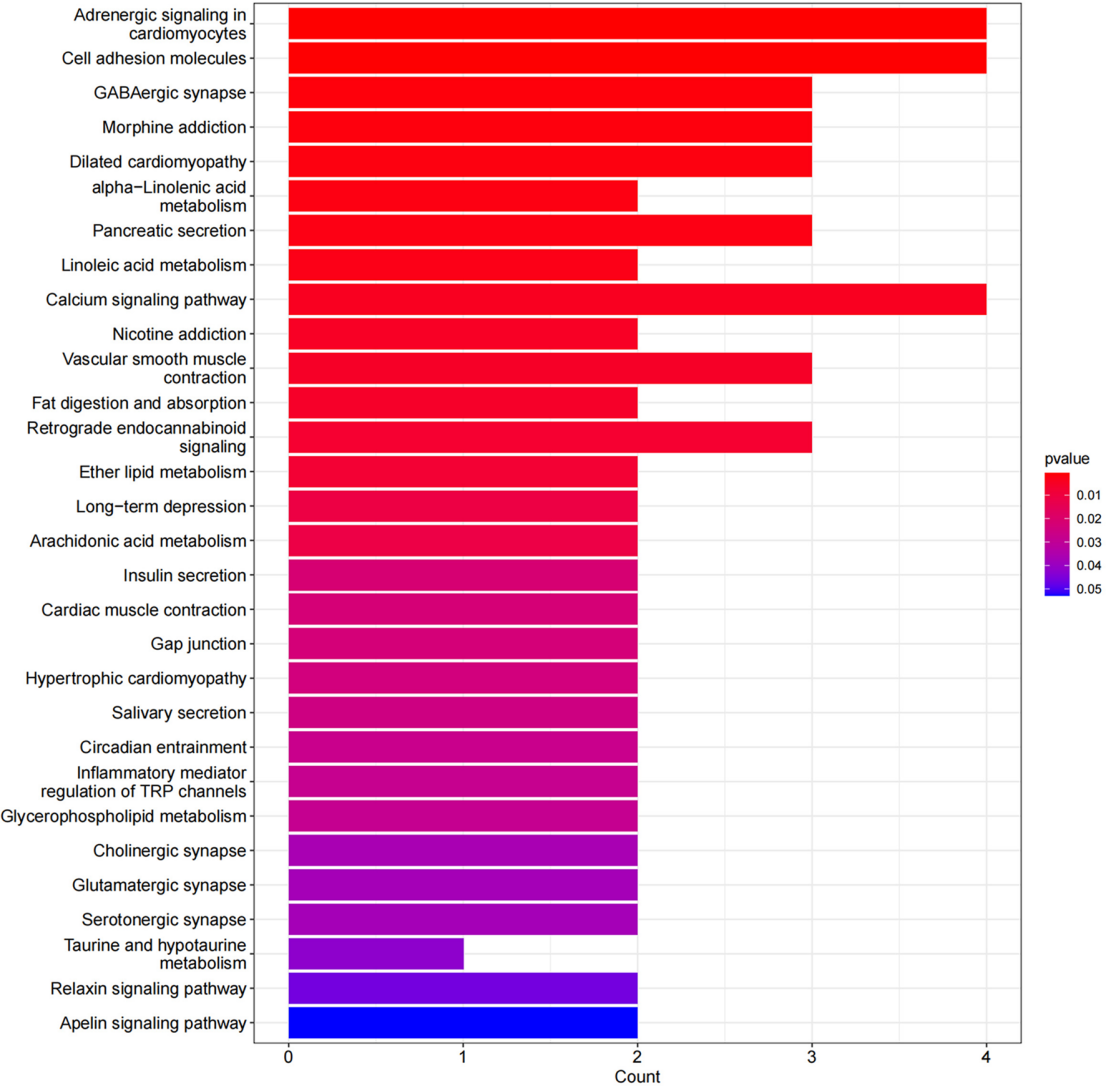
Significantly enriched KEGG pathways of DEGs in HPSCC.
4. PPI analysis: After importing the differentially expressed genes into the STRING database (https://string-db.org/), the output file was imported into Cytoscape software for visualization. The cytoHubba plug-in in Cytoscape was used to calculate the hub genes based on degree. The top five genes were GABRG2, CACNA1A, CNTNAP2, NOS1, and SCN4B, with degrees of 10, 9, 8, 7, and 7. The PPI results are shown in Figs. 5, 6 and 7.

**Figure 5:**
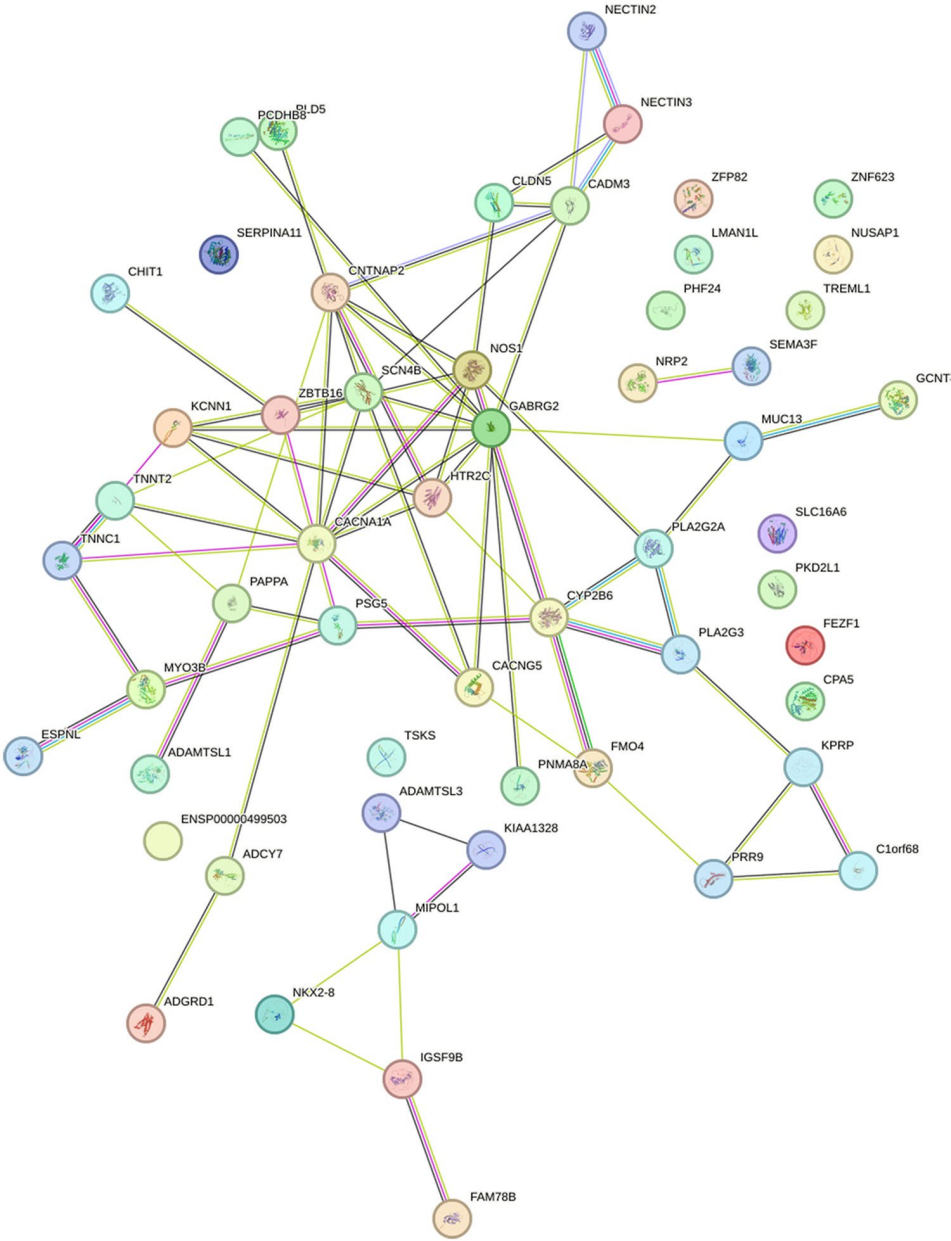
The visualization results of differentially expressed genes in the STRING database.

**Figure 6:**
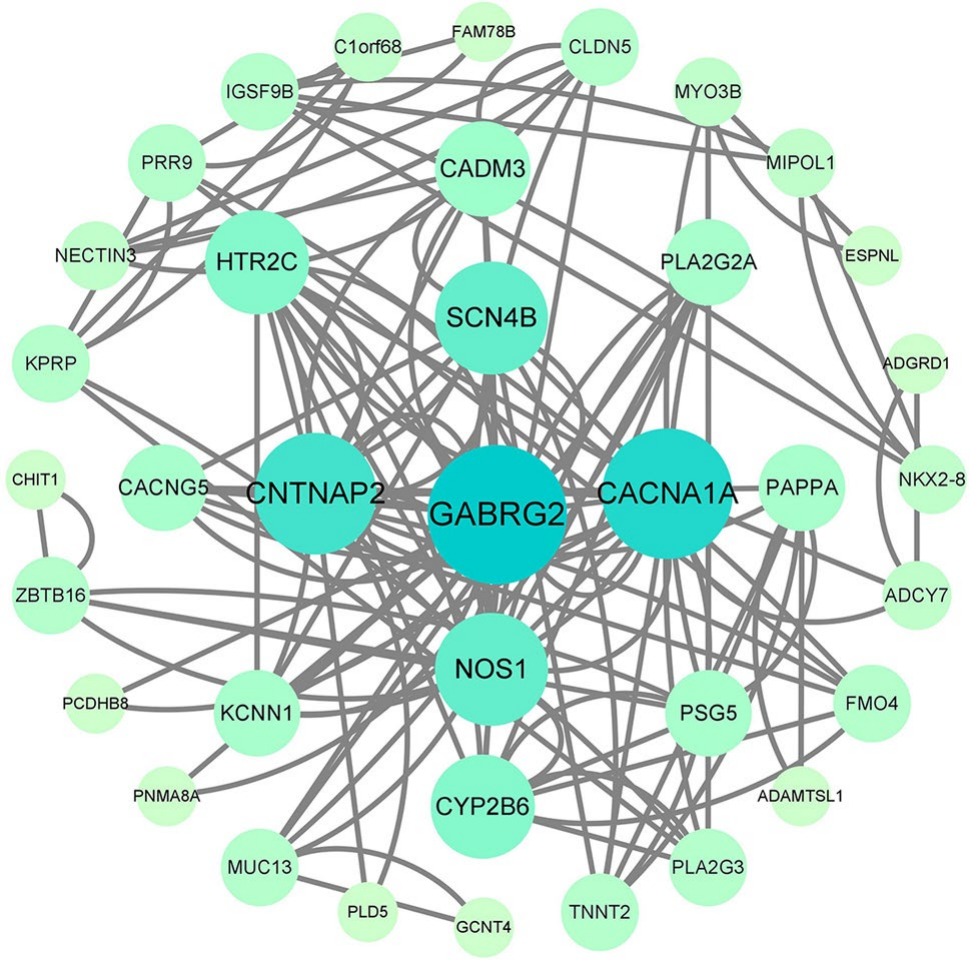
The protein interaction network results in differentially expressed genes, where nodes represent genes and lines represent interactions between two genes. The more lines connected to a node, the greater its connectivity, indicating the importance of the node gene in the network.

**Figure 7:**
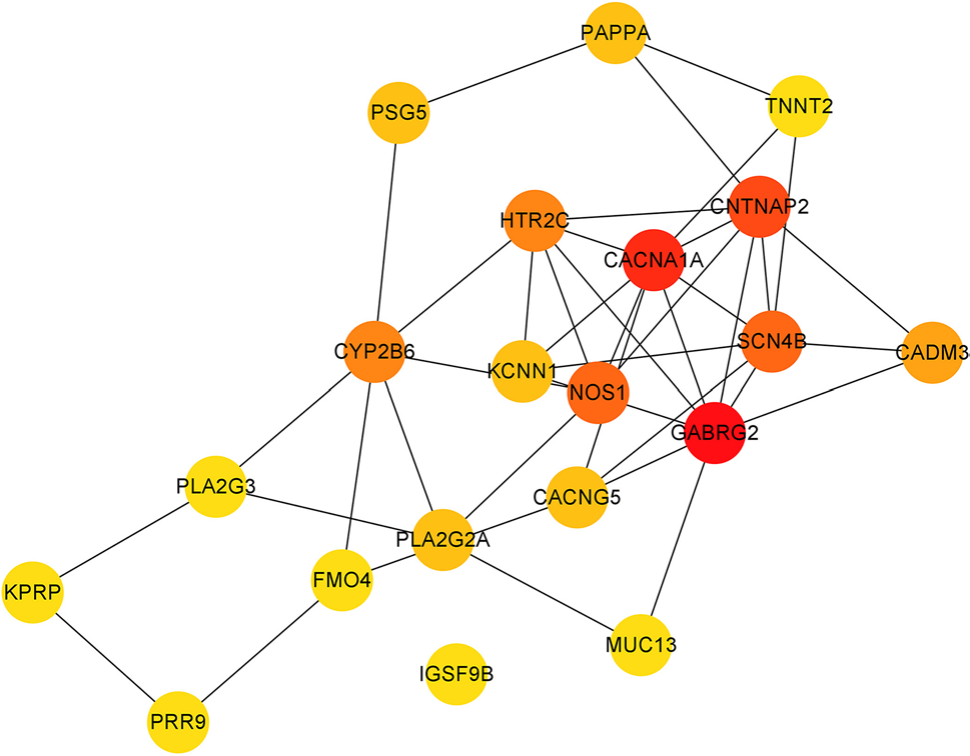
The visualization results of hub genes.
5. Evaluation of hub genes in clinical diagnosis and treatment
  The diagnostic value of these hub genes was evaluated using ROC analysis. The ROC curve is a plot of the true positive rate (sensitivity) against the false positive rate (1-specificity) based on a series of different binary classifications (cut-off values or decision thresholds). The area under the curve (AUC) is used to determine the accuracy of diagnosis. In principle, the diagnostic results do not match the actual situation when the AUC < 0.5, while AUC = 0.5 indicates no effect at all. When 0.5 < AUC < 0.7, 0.7 < AUC < 0.9, and AUC > 0.9, the diagnostic effect is low, medium, and high, respectively, suggesting that GABRG2, CNTNAP2, and SCN4B have high diagnostic value for patients with hypopharyngeal carcinoma combined with esophageal carcinoma, while NOS1 and CACNA1A have moderate diagnostic value. The ROC and nomogram results are shown in Figs. 8 and 9.

**Figure 8:**
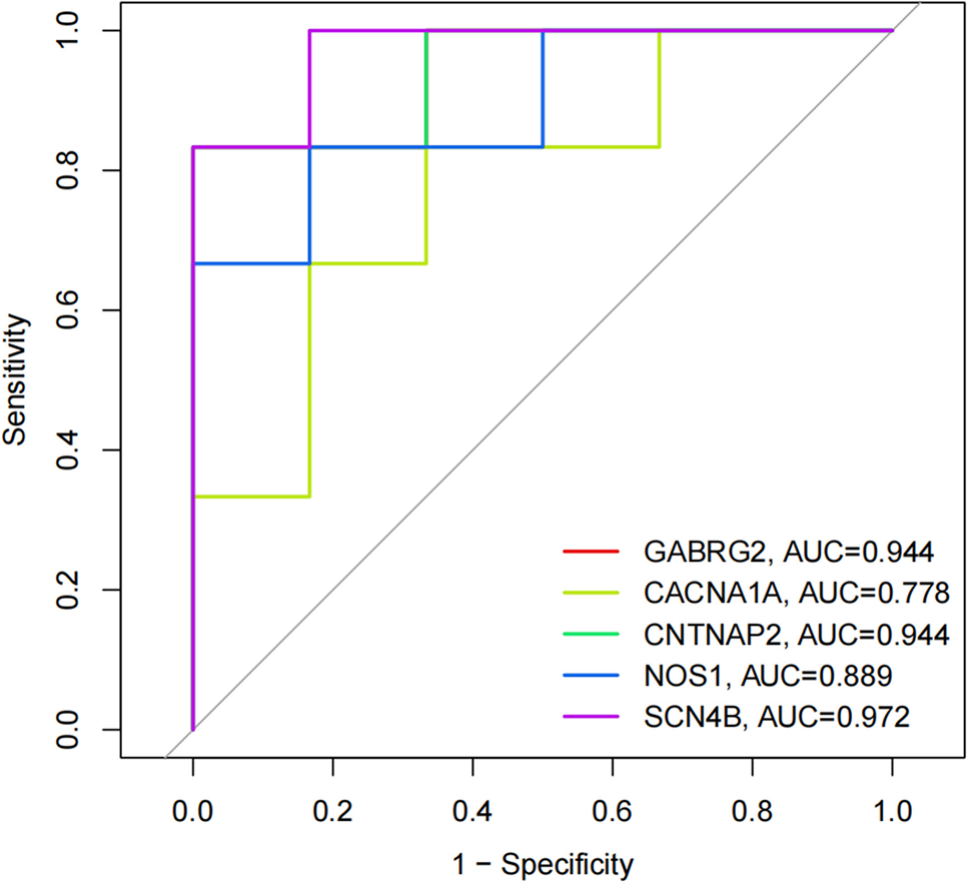
The ROC curve results for the hub genes.

**Figure 9:**
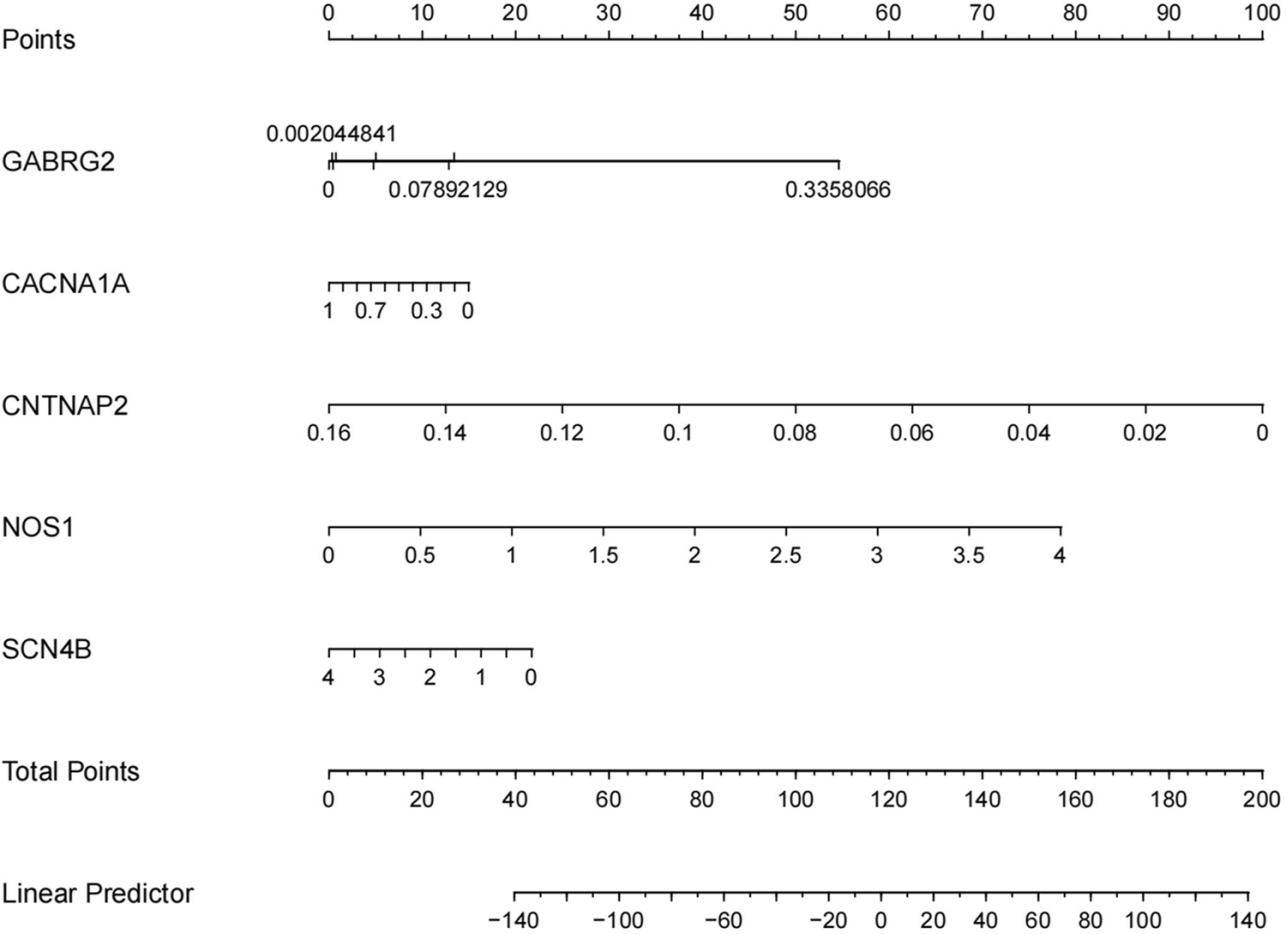
The nomogram for the hub genes. Based on the results of patient genetic testing, corresponding shubs can be obtained for the expression level of each gene. Adding all score hubs can predict the risk of comorbidity of esophageal carcinoma in patients with hypopharyngeal carcinoma.
6. The results of PCR to detect the expression of the six differentially expressed genes with significant differences and relatively high expression levels—LINC00470, CYP2B7P, NKX2-8, CADM3, MIPOL1 and ZFP82 are shown in Fig. 10,* indicates *p* < 0.05, *** indicates *p* < 0.001, and the results are statistically significant, and the detection of the expression is basically the same as the sequencing results. Group A is hypopharyngeal cancer combined with esophageal cancer, Group B is hypopharyngeal cancer.

**Figure 10:**
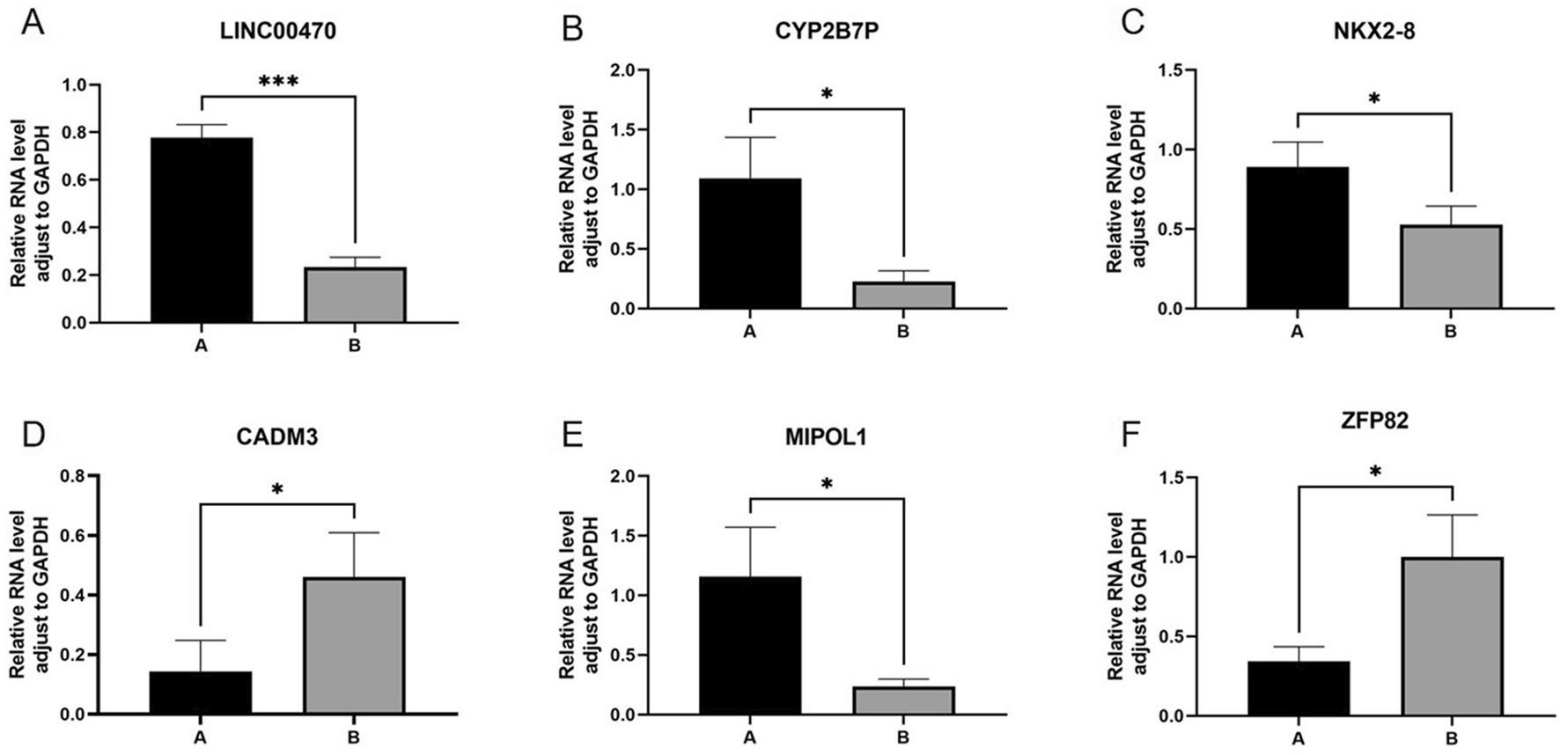
The results of PCR detection of LINC00470, CYP2B7P, NKX2-8, CADM3, MIPOL1 and ZFP82 are shown in Fig. 9, * indicates *p* < 0.05, *** indicates *p* < 0.001, and the results are statistically significant, and the detection of the expression is basically the same as the sequencing results. Group A is hypopharyngeal cancer combined with esophageal cancer, Group B is hypopharyngeal cancer.

## Discussion

The theoretical basis for the occurrence and development of hypopharyngeal carcinoma combined with esophageal carcinoma is widely supported by the field cancerization theory first proposed by Slaughter et al. in 1953 during the analysis of oral squamous cell carcinoma^6^. However, the current mechanism of the occurrence and development of hypopharyngeal carcinoma combined with esophageal carcinoma is not well understood, and various studies have suggested that there may be co-morbid factors in hypopharyngeal carcinoma combined with esophageal carcinoma. Studies have shown that when PD-L1 is used as a predictive biomarker for immunotherapy for hypopharyngeal carcinoma combined with esophageal carcinoma, the immune-related expression of tumor tissues in patients with hypopharyngeal carcinoma combined with esophageal carcinoma is significantly correlated based on PCR and immunohistochemical results^7^. There was a study conducted bioinformatics analysis on independent datasets of hypopharyngeal carcinoma combined with esophageal carcinoma in the GEO database and identified miR-29 as the most frequently interacting miRNA with hub genes^8^. However, the conclusion still needs to be verified by clinically relevant experiments. Another research evaluated the expression of proteins related to hypopharyngeal carcinoma combined with esophageal carcinoma and detected the overexpression of p53 and the deletion of Fhit expression in the relevant tissues by immunohistochemistry^9^. The above studies suggest that there may be common pathogenic genes between hypopharyngeal carcinoma combined with esophageal carcinoma.

In this experiment, we screened the differential genes between hypopharyngeal carcinoma combined with esophageal carcinoma and hypopharyngeal carcinoma based on RNA-seq and verified the results by PCR, which were basically consistent with the results of the gene screening, and then assessed the utility of the hub genes screened by bioinformatics and constructed the prediction model for their utility in clinical diagnosis and treatment. Among them, GABRG2 and CACNA1A were up-regulated, while genes CNTNAP2, NOS1 and SCN4B were down-regulated. These five genes are all related to the occurrence and development of tumors and can be used as hub genes for predicting the risk of patients with hypopharyngeal carcinoma combined with esophageal carcinoma. To the best of our knowledge, there have been no previous studies reporting the association between GABRG2, CACNA1A, CNTNAP2, NOS1, and SCN4B and hypopharyngeal carcinoma combined with esophageal carcinoma. Therefore, these genes may represent new biomarkers or may exhibit gene fusion in hypopharyngeal carcinoma combined with esophageal carcinoma. However, their specific mechanisms in the occurrence and development of hypopharyngeal carcinoma combined with esophageal carcinoma require deeper exploration.

GABA is the most abundant inhibitory neurotransmitter, and its receptor is an important pharmacological target for many antiepileptic drugs.GABRG2 is located on chromosome 5 and encodes the γ2 subunit of GABAAR, which plays an important role in receptor trafficking and post-synaptic membrane aggregation^10^. It is the most common epileptic pathogenic gene among GABAAR subunits^11^. It is abnormally expressed in various cancers such as primary glioblastoma, hepatocellular carcinoma, and colon adenocarcinoma^12–14^. There is relatively little research on GABRG2 in head and neck squamous cell carcinoma. Related studies have found that GABRG2 may regulate laryngeal cancer recurrence by participating in the EMX2OS-miR-124-

CALCA/GABRG2 axis^15^, but the conclusions of this study need to be further verified by relevant molecular experiments. As the gene with the strongest degree of interaction in PPI, the biological role of GABRG2 overexpression in hypopharyngeal carcinoma with esophageal carcinoma deserves further study through in vitro and in vivo research.

CNTNAP2 is located on chromosome 7q35 and encodes a presynaptic type I transmembrane protein CASPR2, which is involved in cell–cell adhesion and synaptic interactions in the nervous system^16^.CNTNAP2 is associated with various neurodevelopmental disorders such as tic-toilet syndrome, intellectual disability, autism spectrum disorder (ASD), and schizophrenia^17^. In aggressive oligodendroglioma, CNTNAP2 expression decreases and is associated with reduced overall survival^18^. In a study of head and neck squamous cell carcinoma, CNTNAP2 has some predictive ability for the sensitivity of laryngeal squamous cell carcinoma to induction chemotherapy^19^.

VGSC (Voltage-gated sodium channel) is a key protein that transmits molecular information.SCN4B (Sodium Voltage-Gated Channel Beta Subunit 4 ) is located on chromosome 11q23.3 and encodes a protein consisting of 228 amino acid residues^20^. SCN4B mutations are associated with a variety of diseases, including cancer, epilepsy, arrhythmia, sudden infant death syndrome, neuropathic pain, and various neurodegenerative diseases^21^. As a tumor suppressor gene, SCN4B plays a role in various cancers. Emeline^22^ and others found that SCN4B expression was downregulated in breast cancer cells and promoted the migration and invasion of breast cancer cells through in vivo and in vitro experimental studies. The expression of SCN4B in the tissues of thyroid cancer patients is down-regulated, and SCN4B expression is an independent predictor of good relapse-free survival in thyroid cancer patients^23^. In prostate cancer cells, miR-3175 expression increases, and SCN4B expression decreases. However, after knocking down miR-3175, cell growth, migration, invasion, and N-cadherin expression, which are related phenotypes, are inhibited, and SCN4B expression is upregulated, suggesting that SCN4B may be a potential mechanism for miR-3175 to promote prostate cancer cell growth and invasion^24^.

Nitric oxide is a signaling molecule synthesized by three subtypes of NO synthase (NOS1, NOS2, and NOS3) and is increased in various cancers and involved in various cancer processes, such as proliferation and migration^25^. NO has a dual effect on tumors. When present at low-moderate concentrations, NO can stimulate various carcinogenic signaling pathways, such as AKT, ERK, and HIF. When present at high concentrations, NO can produce nitrosative stress and stimulate anti-cancer signaling mechanisms, such as p53 and apoptosis pathways^26^. Gesche et al. evaluated relevant tissue samples from 30 patients with primary oropharyngeal squamous cell carcinoma using immunohistochemistry and RT-PCR and found that NOS1 and NOS3 expression significantly increased^27^. NOS1 can participate in the development of various cancers through protein S-nitrosylation, which is a covalent post-translational modification that results in the coupling of NO moieties containing active thiol groups with protein cysteine residues to form S-nitrosothiol (SNO) moieties^28^. NOS1 reduces the recruitment of STAT1-mediated HDAC2 to ISG promoters and promotes lung metastasis of melanoma through the S-nitrosylation of HDAC4-C16/C2^29^. NOS1 can also activate the AKT/mTOR signaling pathway through the S-nitrosylation of PTEN, thereby inhibiting autophagy in nasopharyngeal carcinoma cells^30^.

CACNA1A (Calcium Voltage-Gated Channel Subunit Alpha1 A) is located on the short arm of chromosome 19 at position 13.13 and encodes the channel-forming protein alpha-1A subunit of the P/Q-type voltage-gated calcium channel^31^. This channel mediates Ca^2+^ ion entry into excitable cells and participates in various calcium-dependent biological processes such as muscle contraction, neurotransmitter, and hormone release. CACNA1A mutations are associated with various neurological diseases such as migraine, epilepsy, paroxysmal ataxia, and spinocerebellar ataxia^32^. In primary glioblastoma, CACNA1A expression is upregulated and involved in the development of cancer stem cells (CSC) and the regulation of related signaling pathways^33^. When studying the effect of radiotherapy in patients with oropharyngeal squamous cell carcinoma, Tomoya Kurokawa et al. found that a gene set consisting of eight genes, including CACNA1A, could be used for prediction and had high predictive power^34^.

Combining previous research on five hub genes, we found that the research on detecting the expression of hub genes was basically consistent with our results, and the PCR results also verified the accuracy of our sequencing results. The mechanism of promoting the occurrence of various tumors also suggests that the hub genes are multidimensional in the process of the occurrence and development of hypopharyngeal carcinoma combined with esophageal carcinoma. According to the results of GO and KEGG, the hub genes mainly constitute cell membrane channel proteins such as ion channel complexes, transmembrane transporter complexes, and transporter complexes, and participate in the transmembrane transport and signal transduction of related substances. Ion channels are responsible for the flow of ions across the cell membrane and play a very important role in human physiology, including the regulation of ion homeostasis, electrical excitability, and cell signaling. However, when the expression of ion channels changes, these channels can cause various channel diseases, including cancer. Specific types of ion channels can participate in different stages of tumor progression and mediate the expression of multiple phenotypes of tumors, such as increasing cell heterogeneity by selectively expressing malignant cell clones that support proliferation, migration, or invasion through the selective expression of ion channel types^35^. In head and neck squamous cell carcinoma, voltage-gated K^+^ channels are abnormally expressed and serve as specific markers in the early stages of tumorigenesis and the late stages of disease progression^36^. Studies have shown that the incidence rate of laryngeal cancer in patients with positive dysplasia of Kv3.4 K^+^ channel subunits is significantly increased^37^. When the expression of Kv3.4 is inhibited, it can selectively block the cell cycle in the G2/M phase and then inhibit cell proliferation. In esophageal squamous cell carcinoma, overexpression of the voltage-gated potassium channel subfamily KCNJ15 can lead to increased cell proliferation^38^. These studies suggest that one of the mechanisms underlying the development and progression of hypopharyngeal carcinoma with co-occurring esophageal carcinoma may be the overexpression or suppression of genes that mediate ion channel-related proteins, leading to abnormal protein function or altered expression. In addition to K^+^ ion channels, Ca2^+^, Na^+^, and Cl^-^ channels have also been associated with cancer. The biological role of the aberrant expression of ion channel-associated proteins mediated by hub gene in hypopharyngeal carcinoma combined with oesophageal carcinoma deserves to be further investigated by in vitro and in vivo experiments, and the relevant contents include the upstream and downstream regulatory genes and related protein expression of hub gene, the pathway of regulation and the related biological functions. This will be our research direction in the next stage.

The onset of hypopharyngeal carcinoma is insidious, and it often progresses to the middle and late stages when diagnosed, with poor prognosis. When combined with a second primary cancer, it can seriously affect the patient's prognosis and survival. Currently, the main method for determining whether hypopharyngeal carcinoma is associated with a second primary cancer of the esophagus is NBI staining endoscopy combined with biopsy. The procedure often causes patients to experience significant pain, and due to the specific anatomical location of the hypopharynx and esophagus, there is a risk of human error during the operation, resulting in missed detection. Therefore, from an objective perspective, molecular markers related to gene detection in patients' blood or other secretions will provide better-individualized treatment options for early detection, diagnosis, and prediction of hypopharyngeal carcinoma patients with or without esophageal carcinoma. This study screened the differentially expressed genes between hypopharyngeal carcinoma with and without esophageal carcinoma by gene sequencing and validated the sequencing results through QRT-PCR experiments. We further used bioinformatics analysis to identify the hub genes of hypopharyngeal carcinoma with or without esophageal carcinoma, suggesting that GABRG2, CACNA1A, CNTNAP2, NOS1, and SCN4B genes may be potential molecular markers for indicating the risk of hypopharyngeal carcinoma co-occurring with esophageal carcinoma. This study will improve the detection rate of precancerous lesions in the esophageal region, which is beneficial for the early diagnosis and treatment of hypopharyngeal carcinoma with or without esophageal carcinoma, as well as the monitoring and follow-up of hypopharyngeal carcinoma after treatment. However, there are still shortcomings in this experiment, such as the small sample size selected, the lack of basic research on the specific mechanisms of hub genes in the occurrence of hypopharyngeal carcinoma with or without esophageal carcinoma, and the potential differences in genes between patients from different regions and ethnic groups. It is unclear whether the diagnostic value of hub genes for hypopharyngeal carcinoma with or without esophageal carcinoma is consistent with the actual clinical situation. Therefore, we will adopt a multi-center study to supplement the number of cases to obtain more data, so that our conclusions are more accurate, to exclude regional and ethnic differences. In the follow-up study, we will add genomic analysis and comparison between normal mucosal tissue and oesophageal lesions to validate the results of our current study and make our conclusions more scientific and accurate.We will further improve cell and animal experiments to explore the mechanisms of hub genes in the occurrence of hypopharyngeal carcinoma with or without esophageal carcinoma. We will adopt more detailed follow-up strategies to fully understand the changes in the condition of hypopharyngeal carcinoma patients. We hope to further improve and refine these problems and deficiencies through subsequent experiments.

## Data Availability

The original contributions presented in the study are included in the article Material. Further inquiries can be directed to the corresponding authors.

## Abbreviations

HPSCC: Hypopharyngeal squamous cell carcinoma
SPM: Second primary malignancies
GO: Gene ontology
KEGG: Kyoto encyclopedia of genes and genomes
PPI: Protein–Protein interaction
BP: Biological processes
CC: Cellular components
MF: Molecular functions
GAPDH: Glyceraldehyde-3-phosphate dehydrogenase
AUC: Area under curve
GABA: γ-Aminobutyric acid
GABRG2: Gamma-Aminobutyric acid type A receptor subunit Gamma2
GABAAR: γ-Aminobutyric acid type A receptor
CNTNAP2: Contactin Associated Protein 2
VGSC: Voltage-gated sodium channel
SCN4B: Sodium voltage-gated channel beta subunit 4
CACNA1A: Calcium voltage-gated channel subunit Alpha1 A
